# Fertility Preservation in Endometriosis Patients: Anti-Müllerian Hormone Is a Reliable Marker of the Ovarian Follicle Density

**DOI:** 10.3389/fsurg.2017.00040

**Published:** 2017-07-25

**Authors:** Elisabetta Garavaglia, Cinzia Sala, Gianluca Taccagni, Michela Traglia, Caterina Barbieri, Stefano Ferrari, Massimo Candiani, Paola Panina-Bordignon, Daniela Toniolo

**Affiliations:** ^1^Obstetrics and Gynaecology Unit, San Raffaele Scientific Institute, Milan, Italy; ^2^Genetics of Common Disorders, Division of Genetics and Cell Biology, San Raffaele Scientific Institute, Milan, Italy; ^3^Pathology Unit, San Raffaele Scientific Institute, Milan, Italy; ^4^Reproductive Sciences, Division of Genetics and Cell Biology, San Raffaele Scientific Institute, Milan, Italy

**Keywords:** ovarian reserve, anti-Müllerian hormone, follicle density, ovarian tissue cryopreservation, fertility preservation

## Abstract

**Objective:**

To analyze the ovarian reserve *via* measurement of follicular density and anti-Müllerian hormone (AMH) in endometriosis patients participating to a clinical program of cortical ovarian cryopreservation.

**Design:**

Retrospective analysis of serum AMH levels and prospective investigation of ovarian follicle number.

**Setting:**

University Hospital.

**Patient(s):**

Two hundred and two women with endometriosis and 400 controls.

**Intervention(s):**

Blood samples and ovarian biopsies.

**Main outcome measure(s):**

Correlation of serum AMH levels and the number of non-growing follicles in the biopsied cortical tissues in endometriosis and control subjects, including age, type of AMH kit, and the laboratory performing the analysis as covariates.

**Result(s):**

AMH levels were shown to decrease with age in untreated endometriosis patients (*P* < 1.0 × 10^−5^) but they were significantly lower in endometriosis compared to controls only in patients over 36 years old (*P* = 2.7 × 10^−4^). The AMH decrease was faster in endometriosis compared to controls (beta = 0.27, *P* = 4.0 × 10^−4^). Primordial follicle number decreased with the reduction of AMH levels in both cases and controls (beta = 0.3; *P* = 0.04).

**Conclusion:**

AMH is a reliable marker of ovarian reserve in endometriosis patients, and it can predict follicular density in women undergoing ovarian tissue cryopreservation.

## Introduction

Fertility preservation represents a significant clinical challenge for the biomedical community. Initially developed for oncological patients (1), approaches to fertility preservation have been extended to women at risk to develop premature ovarian insufficiency (POI) as a consequence of genetic or environmental causes and/or gynecological treatments (2).

Available options for fertility preservation for adult women include the cryopreservation of oocytes, which, thanks to the development of vitrification protocols, allows the achievement of fertilization rates comparable to those with fresh gametes (3, 4). However, this approach cannot be applied to young women who have not yet reached sexual and psychological maturity, to patients who need to start an immediate anticancer treatment, or to women with hormone-sensitive malignancies. In these cases, cryopreservation of the ovarian cortical tissue could be proposed (5). Although about 60 babies were born so far after ovarian tissue transplantation (6), this method is still considered an experimental technique. Yet, new technologies for *in vitro* maturation of oocytes collected from the ovarian cortex during laparoscopic surgery have recently opened new horizons (7). However, low follicular density may limit the success of the fertility restoration and the identification of presurgical reliable markers of ovarian follicle density could help to avoid useless surgeries and, at the same time, give indications of the optimal age for tissue collection. To date, the most used non-invasive tests are the ovarian antral follicle count (AFC), evaluated by ultrasound, and serum anti-Müllerian hormone (AMH) (8) both considered predictive in assisted reproductive treatment (ART) or for age of menopause (9, 10).

Among the many iatrogenic and pathological conditions that compromise ovarian function, endometriosis, a sterile inflammatory disease characterized by growth of endometrial tissue at ectopic location, may represent a cause of early menopause, as indicated by its association with infertility in more than 50% of affected patients (11, 12). The chronic clinical course of endometriosis, associated with its common bilateral involvement, and the frequently repeated surgical interventions suggest that ovarian cryopreservation might represent a promising option for fertility preservation in affected women.

The primary goal of this study was to evaluate presurgical serum AMH levels in a cohort of endometriosis patients undergoing ovarian cortex resections for fertility preservation at our institution in the years 2011–2014, compared to a large control group, and then to test the hypothesis that the preoperative AMH values correlated with the individual follicular density.

To our knowledge, this is the first study that analyzed ovarian reserve through combined measurement of follicular density and serum AMH levels in the same endometriosis subjects. Even though a limited sample size was analyzed, we showed that AMH might be used as a marker of ovarian reserve in endometriosis cases.

## Materials and Methods

### Human Subjects

Two hundred and two women with endometriosis undergoing surgery for the first time were included in the study. The majority (156; 77.2%) presented with ovarian endometriosis, of which 35 (22.4%) had bilateral and 121 (77.6%) had unilateral endometriosis. All patients were in premenopausal period, with a mean age of 34.7 ± 5.9 years (19–48 age range). The AMH dosage was performed the day before surgery. Ovarian biopsies were collected from a subgroup of 25 patients at the beginning of the surgery to avoid thermal, vascular, chemical, and mechanical damage of the ovaries. Biopsies were collected with microscissors with cold blade from healthy ovarian cortex, either from the non-affected ovary or, in cases of bilateral disease, far from the cyst. Tissue samples of about 4 mm × 4 mm were transferred to the pathology laboratory in formaldehyde. For histochemistry analysis, samples were randomly collected far from the affected site, as a previous study reported that ovarian biopsies sampled at random show an error of 0.5% in estimating the non-growing follicles (13).

Control biopsies were taken from 33 patients undergoing surgery for causes different from endometriosis. Indications for surgery were uterine leiomyomas with menorrhagia (*n* = 14), ovarian cryopreservation for extra-gynecological oncologic reasons (*n* = 4), infertility due to tube obstruction (*n* = 7), and non-endometriotic cysts (*n* = 8).

Controls for serum AMH levels were subjects from two different populations of premenopausal women all of Italian origin. They included 200 women randomly selected from the Italian Network of Genetic Isolates/Val Borbera population (14), a normal population of North West Italy, and 200 randomly selected women from a cohort undergoing ART for male infertility at the Obstetrics and Gynecology Unit, San Raffaele Scientific Institute.

The study including the overall plan and the informed consent form was reviewed and approved by the institutional review boards of the San Raffaele Scientific Institute.

### Tissue Preparation and Follicle Counts

**Figure 1:**
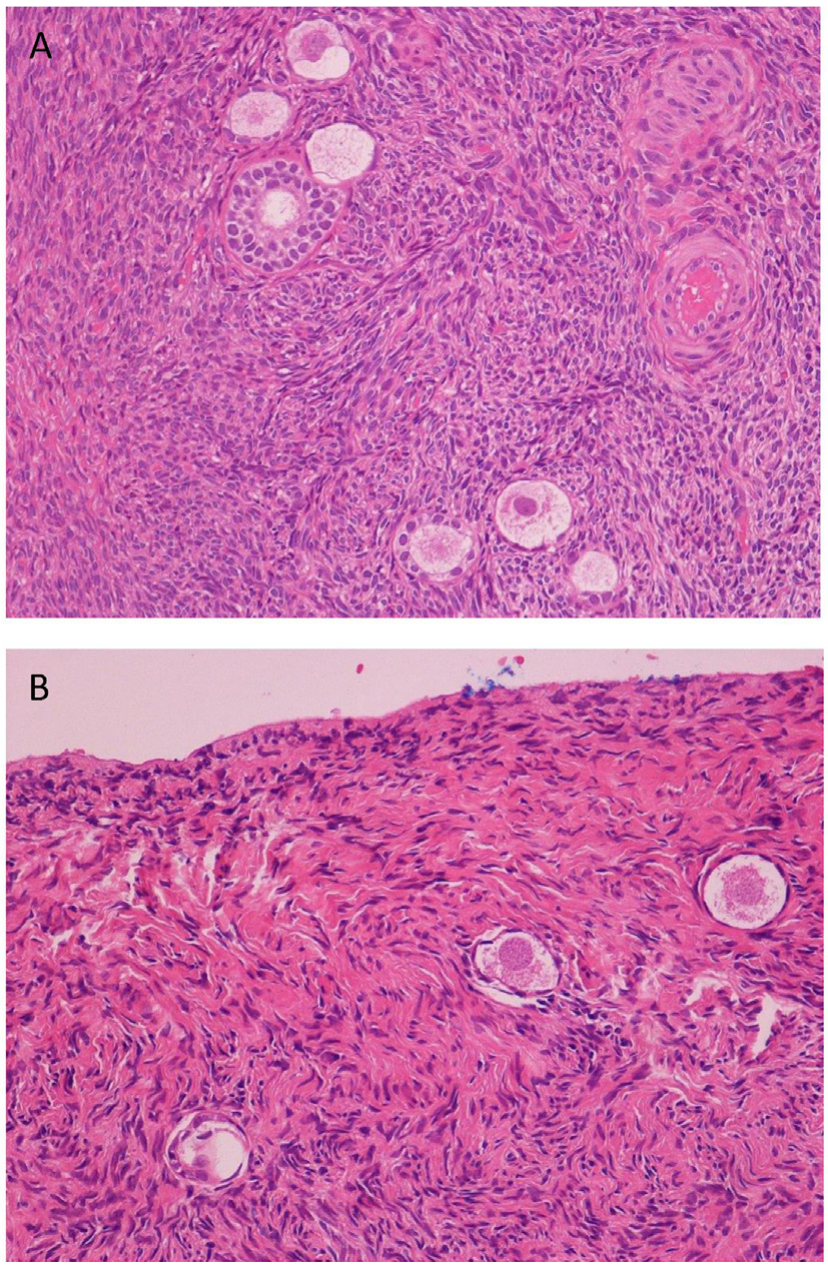
Histological analysis of cortical ovarian strips. **(A)** Hematoxylin/eosin staining shows primordial, early, and mature primary follicles of one representative ovarian cortical strip of a control (125×). **(B)** Hematoxylin/eosin staining shows a few primordial follicles (severe ipotrophia for age of the patient) in a fibrosclerotic tonaca albuginea of one representative cortical strip of an endometriosis patient (200×).

During the paraffin wax embedding, each ovarian biopsy was marked by Indian ink on the inner part in order to obtain a right orientation from ovarian surface to deep cortex. Paraffin block was then cut perpendicularly to ovarian surface into 5-μm sections, at 50-µm intervals to avoid the overcounting of the same follicle (primordial and primitive follicles do not exceed this size). Sections were then stained with hematoxylin and eosin. All sections were analyzed using an Olympus microscope at 200× magnification. The developmental stages of follicles were defined according to Gougeon’s protocol (1986), which defines that the primordial follicle is a structure containing the primary oocyte surrounded by a single flat cells stratum, while the primitive follicle is an element assembled by the primary oocyte surrounded by the *zona pellucida* and by 1–2 cubical cells of the granulosa tissue (Figures 1A,B). The follicle density was determined by dividing the number of follicles counted in 10 non-adjacent fields of 1 mm^2^ each by the volume of tissue analyzed (0.5 mm^3^) and expressed as number of follicles per mm^3^.

### Hormone Assay

Serum AMH levels were measured in cases and in the two control cohorts in the course of several years by two types of commercially available immunoenzymatic assays: the GenII ELISA (Beckman Coulter) and the EIA AMH/MHS kit (Immunotech, Beckman Coulter). The limit of detection (LOD) of both assays was 0.14 ng/ml. For participants with measurements below the LOD, values were replaced with LOD/√2 (15). AMH was detected in 8% of cases and 2.5% of controls. Intra-assay and inter-assay coefficients of variation were 12.3 and 14.2%, respectively.

### Statistical Analysis

In-house R 3.1.1 scripts (http://www.r-project.org) were used for descriptive and inferential statistics analyses of follicles counts and the levels of AMH in the total sample (*N* = 602). For each measurement, outlier values were excluded using a 3 SD threshold based on Shapiro–Wilk normality tests assessing the normality of the distributions. After outliers exclusion, we analyzed 588 AMH (201 cases and 387 controls) and 57 follicle counts (24 cases and 33 controls). Then, follicle counts were square root transformed to normalize. AMH dosage was transformed with a rank-based inverse normalization using the quantile R function qnorm (*p*, μ, σ) that converts proportions to quantiles. The continuous distributions of each transformed trait were represented by density() R function. The R wilcox.test() function and/or t.test() function were used to estimate significant difference in the median and/or mean of case/control population and in young and old (≤36 and >36 years old). The glm() R function was used to fit generalized linear models in regression analyses, and ggplot2 library was used for exploratory data analysis and plotting results.

## Results

### AMH Levels Were Progressively Reduced with Age in Untreated Endometriosis Patients

Serum AMH values of 201 untreated endometriosis patients and 387 normal women were included in the analysis. The mean of AMH level was lower in cases compared to controls (Table 1). Since AMH levels were measured using two different kits by two independent laboratories, we tested the statistical dependence of AMH and the two confounding factors, kit and laboratory, in addition to age. All variables showed significant correlations (*P* < 1.0 × 10^−5^) assessed using the Spearman’s rank correlation test, and were therefore included as covariates in all the analyses. Regression analysis, after the exclusion of the confounding effect of the whole set of covariates, showed that the median of the AMH residual levels was significantly lower in cases [median = −0.17 (−1.97 to 2.99)] compared to controls [median = 0.06 (−3.03 to 2.67)] (*P* = 1.0 × 10^−3^).

**Table 1:** Serum AMH concentrations in cases and controls.

	Number	Age (years)^a^	AMH (ng/ml)^a^
Cases	201	34.72 ± 5.93	1.786 ± 1.87
Controls	387	35.30 ± 6.36	2.928 ± 2.28

*^a^Mean ± SD*.

*AMH, anti-Müllerian hormone*.

It has been estimated that women older than 36 years present a significant depletion of the ovarian reserve (16, 17). When the threshold of 36 years of age was used to subdivide the population in two groups, only >36 year-old cases had a significant decrease in AMH residual levels compared to controls: cases median of residues = −0.87 (−1.82 to 2.58) versus controls median of residues = −0.25 (−2.30 to 2.57) (*P* = 2.7 × 10^−4^).

**Figure 2:**
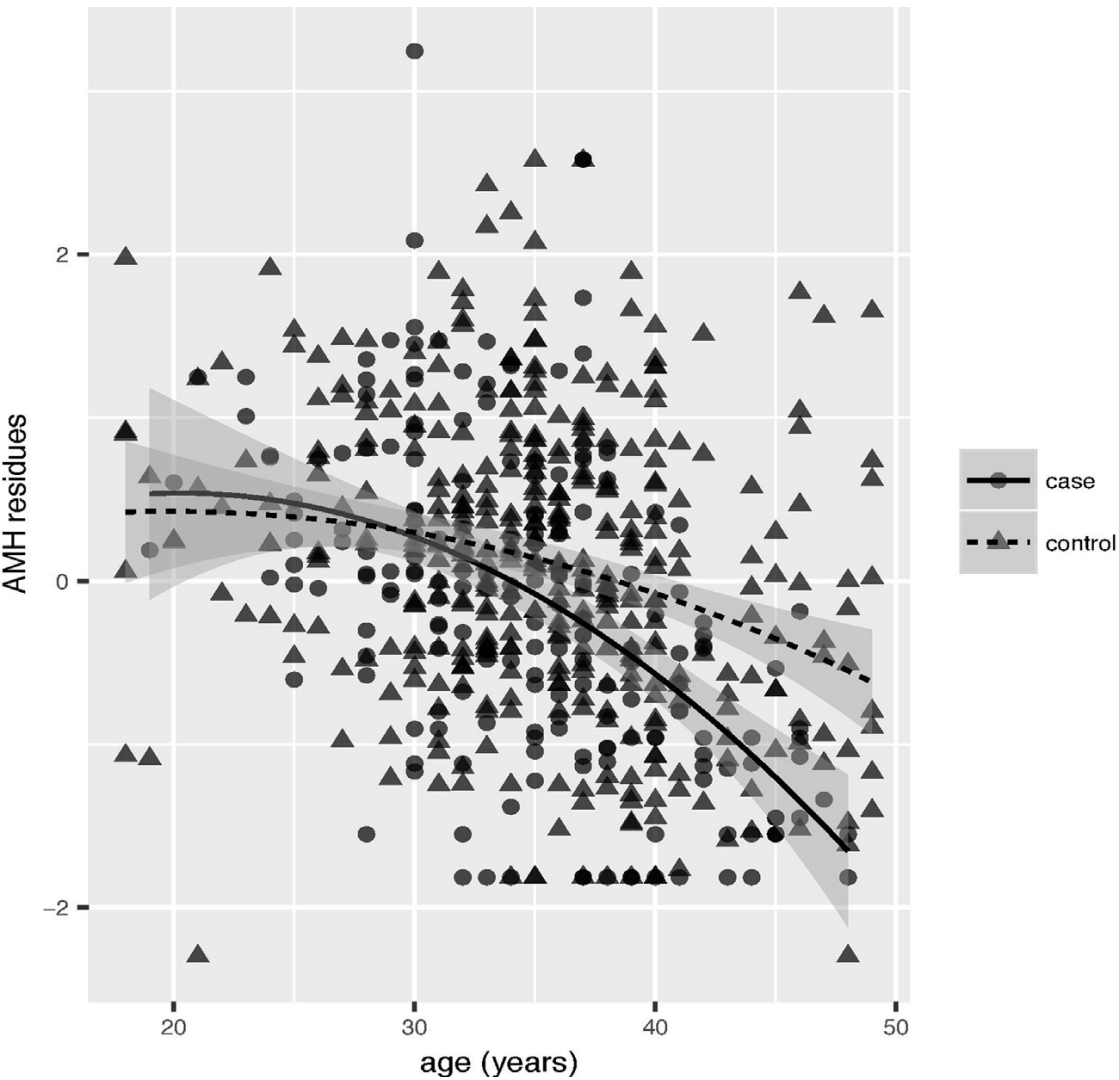
Multiple regression analysis of anti-Müllerian hormone (AMH) and age. AMH kit and laboratory were included as covariates. Scatter plot of the correlation between AMH residues and age in cases (solid circles) and controls (solid triangles). Linear interpolation for cases (solid lines) and controls (dashed lines) together with confidence intervals (gray shadowed area) are reported.

To better describe the decrease of AMH levels with age in endometriosis patients, we performed a multiple regression analysis of AMH and age including all the above covariates. Serum AMH levels decreased with increasing age in patients and in controls as expected, but the decrease was faster in endometriosis cases (Figure 2). To conclude that the effect of age was different in cases and controls, the interaction between age and affection status was included in the analysis: the interaction was highly significant (beta = 0.27, *P* = 4.0 × 10^−4^). The faster decrease of the AMH residual levels with age was further confirmed when either considering all ovarian endometriosis cases (*n* = 155), or separating bilateral (*n* = 35) from unilateral (*n* = 120) cases (data not shown).

### Primordial Follicle Counts Correlated with the Decrease in AMH Levels in Both Cases and Controls

Ovarian biopsies were performed in a subset of 24 endometriosis cases and 33 controls (Table 2). Counts of primordial follicles did not show significant differences between cases and controls. Primordial follicle numbers were slightly lower in endometriosis cases but the difference was not statistically significant even if only older cases (>36 years) were considered. As expected, the follicle number decreased with age both in endometriosis cases and controls (beta = −0.19; *P* = 2.15 × 10^−5^).

**Table 2:** Primordial and primary follicle counts from ovarian biopsies in cases and controls.

	Number	Primordial^a^	Primary^a^
Cases	24	12.25 ± 16.48	7.9 ± 7.35
Controls	33	14.42 ± 18.36	10.42 ± 12.16

*^a^Mean ± SD*.

To test our primary hypothesis, that a correlation between AMH levels and follicle numbers could be established in endometriosis patients, we performed a regression analysis of AMH and follicle numbers including age and kit/laboratory as covariates, as discussed above. Considering the entire dataset, including the endometriosis patients, a statistically significant correlation was observed between AMH residual levels and the numbers of primordial follicles (beta = 0.3; *P* = 0.04): lower AMH residual levels corresponded to lower numbers of primordial follicles. The beta of the regression curve was higher in cases, suggesting a faster depletion of primordial follicles in endometriosis patients (Table S1 in Supplementary Material); however, due to the small number of individuals available when the controls were analyzed separately or affection status was included in the analysis, the regression was not statistically significant.

## Discussion

In the last 10 years, oocyte and ovarian cortical tissue cryopreservation have become valuable options to restore fertility and improve quality of life of patients with early menopause. Ovarian cryopreservation, which represents the only choice for prepuberal patients (5), is strictly dependent on the presence of an adequate number of follicles in the surgically removed cortical ovarian strips. As this cannot be merely predicted from the chronological age, presurgical ovarian reserve tests are dearly needed to avoid useless surgery (18).

Anti-Müllerian hormone is the best available marker of ovarian reserve and an indicator of response to gonadotropin stimulation (9). Despite its variability, the threshold value of 1.28 ng/ml, identified as an indicator of sufficient immature oocyte yield (19), is currently used in *in vitro* fertilization. It has been shown repeatedly that AMH levels decrease in endometriosis patients after surgery (20, 21), but only small studies have been published on presurgical serum AMH level, in patients of Asian origin to determine whether AMH is a reliable marker also for endometriosis-associated infertility and it could be used to predict follicular density for fertility preservation.

We here report a study of AMH levels and ovarian reserve in 201 untreated endometriosis patients of Italian origin undergoing surgery for the first time. The results showed that AMH levels before surgery were significantly lower in endometriosis cases compared to controls (*P* = 1.0 × 10^−3^), in agreement with a similar analysis in a cohort of patients from Korea (22). Interestingly, by splitting our cohort into two different age groups, we showed that AMH levels might account for a difference between cases and controls only in women older than 36 years. When we analyzed older patients separately from younger patients, we showed that only in older women the mean value of AMH levels were significantly different in cases and controls (*P* = 2.7 × 10^−4^). Accordingly, serum AMH levels decreased faster with increasing age in endometriosis cases compared to controls. The difference between cases and controls was statistically significant (*P* = 4.0 × 10^−4^). The endometriosis localization did not seem to influence the observed faster decrease of the ovarian reserve in endometriosis patients, suggesting that it may be the general inflammation status, free radicals, hyperoxidation, and iron deposits or the genetic alterations that predispose to endometriosis to influence the ovarian reserve and possibly also worsen follicle quality in endometriosis (23).

Anti-Müllerian hormone levels and ovarian follicle numbers have been shown to correlate in previous studies. We now show that they appear directly correlated also in our study of endometriosis patients. Despite the small number of individuals available, we could correlate the number of primordial follicles and the AMH levels in the same individual, giving our study a greater sensitivity than similar studies (10). The numbers of primordial and primary follicles were slightly lower but not significantly different in endometriosis cases, and the comparison between young (≤36 years) and older (>36 years) women did not change the results. Interestingly, by analysis of the entire dataset, we significantly associated AMH levels with the number of primordial follicles (beta = 0.3; *P* = 0.04): as expected, lower AMH level corresponded to a lower number of primordial follicles. When the affection status was included in the analysis, the difference between cases and controls was not statistically significant (beta = −0.05; *P* = 0.87). The lack of significance could be due to the small number of individual pieces of ovarian cortex studied so far, as it is well known that follicle density may vary through the whole ovarian cortex (24). Indeed, analysis of larger dataset could add more power and demonstrate even small differences. It is interesting to note here that, when cases and controls were analyzed separately, the effect (beta) of the AMH level on the number of primordial follicles was higher in cases compared to controls. This suggests a faster decline of the number of primordial follicles associated with a faster decrease of AMH levels, and thus a direct effect of endometriosis on ovarian reserve.

The analysis of a larger cohort of patients could definitively add significance to the study, although our data already indicate that AMH represents a reliable marker of ovarian reserve in untreated endometriosis patients. Importantly, our study shows that AMH levels should be evaluated early during disease progression, to take into account the accelerated follicular depletion, and thus justify ovarian cryopreservation.

## Ethics Statement

This study was carried out in accordance with the recommendation and the guidelines of the Ethic Committee of San Raffaele Scientific Institute with written informed consent from all subjects. All subjects gave written informed consent in accordance with Declaration of Helsinki. The protocol was approved by the Ethic Committee of San Raffaele Scientific Institute.

## Author Contributions

EG has conceived the project. EG, SF, and MC performed the ovarian biopsies. GT performed the follicular count on each biopsy. CS, MT, and CB performed the statistical analyses. EG, DT, PP-B, and CS prepared the manuscript. All authors work at the San Raffaele Scientific Institute, Milan, Italy.

## Conflict of Interest Statement

The authors declare that the research was conducted in the absence of any commercial or financial relationships that could be construed as a potential conflict of interest.
