# Supplementary material for: Fertility Preservation in Endometriosis Patients: Anti-Müllerian Hormone Is a Reliable Marker of the Ovarian Follicle Density
Source: Front Surg. 2017 Jul 25;4:40. doi: 10.3389/fsurg.2017.00040 (PMC5524724; doi:10.3389/fsurg.2017.00040)
Supplement: Supplementary file 1 [file Table_1.docx]

Supplementary Table I. Regression analysis of primordial follicles and AMH.

|  | Number | Covariate | p-value | beta | SE |
| --- | --- | --- | --- | --- | --- |
| All | 46 | AMH Kit + age | 0.0434 | 0.2997 | 0.144 |
| cases | 21 | AMH Kit + age | 0.0658 | 0.3718 | 0.19 |
| controls | 25 | AMH Kit + age | 0.2 | 0.318 | 0.241 |
